# Evaluation of Antidiabetic Activity of *Ajuga integrifolia* (Lamiaceae) Root Extract and Solvent Fractions in Mice

**DOI:** 10.1155/2020/6642588

**Published:** 2020-12-22

**Authors:** Molalegn Alene, Mohammedbrhan Abdelwuhab, Assefa Belay, Taklo Simeneh Yazie

**Affiliations:** ^1^Department of Pharmacology, College of Medicine and Health Sciences, University of Gondar, P.O. Box 196, Gondar, Amhara, Ethiopia; ^2^Pharmacology Unit and Research Team, Department of Pharmacy, College of Health Sciences, Debre Tabor University, P.O. Box 272, Debre Tabor, Amhara, Ethiopia

## Abstract

Traditional healers and the community have used the roots of *Ajuga integrifolia* for the treatment of diabetes mellitus. It is not scientifically validated for its antidiabetic activity previously. Therefore, the objective of the present study was to determine the hypoglycemic and antidiabetic activity of *Ajuga integrifolia*. *Ajuga integrifolia* roots' crude extract and solvent fractions were prepared. The doses of 100 mg/kg, 200 mg/kg, and 400 mg/kg of crude root extract and solvent fractions were used on normoglycemic, oral glucose loaded, and streptozotocin-induced diabetic mice models to determine their hypoglycemic and antihyperglycemic activities. The crude extract and solvent fractions' effect on bodyweight was also evaluated on streptozotocin-induced diabetic mice. A standard drug in all cases was glibenclamide (5 mg/kg), and the blood glucose level was measured by using a glucose meter. Data analysis was performed by using Statistical Package for Social Sciences version 21. One-way analysis of variance followed by Tukey's post hoc multiple comparison test was used to analyze the data. *p* value < 0.05 was considered statistically significant. Hydromethanolic crude extract and its aqueous fraction of *Ajuga integrifolia* root showed a considerable blood glucose lowering activity at all doses. Both the repeated daily doses of the crude extract and the repeated daily doses of the aqueous fraction of *Ajuga integrifolia* root extract revealed the similar effect in lowering the fasting blood glucose level in streptozotocin-induced diabetic mice models. It was also found that groups treated with the *Ajuga integrifolia* at the doses of 200 mg/kg and 400 mg/kg showed significant (*p* < 0.05) bodyweight improvement at the 14^th^ day of treatment compared to the respective baseline bodyweight, and the diabetic control group showed significant (*p* < 0.01) reduction in bodyweight at the 14^th^ day compared to the baseline. This study revealed that crude extract and solvent fractions of *Ajuga integrifolia* root possess significant antidiabetic activity which supports its traditional use for the treatment of diabetes mellitus.

## 1. Background

Diabetes mellitus (DM) is a cluster of metabolic diseases occurred as a result of insulin deficiency, insulin resistance, or both that result in hyperglycemia [1]. Long-term damage and failure of the eyes, kidneys, nerves, heart, and blood vessels were evident in chronic hyperglycemia [2]. Development of DM involves different pathogenic processes, and these range from autoimmune destruction of the pancreatic *β*-cells that result in absolute insulin deficiency to abnormalities that result in insulin resistance. The basis of the abnormalities in the metabolism of carbohydrate, fat, and protein in diabetes is insufficient action of insulin on target tissues [3, 4]. The projected number of people aged 20–79 years with diabetes was 463 million worldwide in 2019. The worldwide prevalence of DM is estimated to rise to 578 million in 2030. The prevalence of diabetes in this age group was expected to be increased by 143% in Africa from 2019 to 2045, which is higher than other parts of the world. Diabetes and its complications caused significant deaths which are estimated to be 4.2 million in 2019. The annual worldwide health cost on diabetes is estimated to be United States Dollar (USD) 760 billion. It is expected that cost will be USD 825 billion by 2030 and USD 845 billion by 2045 [5].

Management of DM includes diet modification, exercise, weight loss, oral hypoglycemic agents, and insulin; however, none of them can treat the underlying cause of the disease nor can they cure the illness consequently [2, 3, 6]. The limitation of currently available drugs in terms of safety, efficacy, and cost warrants the development of new antidiabetic drugs from plant-derived compounds which are more efficacious, safer, and easily accessible [2, 5]. Worldwide, more than 1000 plants have been known as efficacious in the treatment of DM. However, fewer than half of these plants or plant extracts have been scientifically validated for their claimed use [7, 8]. Lamiaceae is one of the plant families that have hypoglycemic activity. *Ajuga integrifolia* is an herb belonging to Lamiaceae family under genus *Ajuga* and is locally known as “Tut Astel” and “Akorarach” in Amharic and “Harmegusa” in Oromifa in various parts of Ethiopia [9–11]. In Ethiopia, *Ajuga integrifolia* occurs in different regions including Amhara, Southern Nation, Nationalities and People (SNNP), Tigray, and Oromia [12]. The aqueous and occasionally alcohol infusion of the fresh or dried leaves or root of the *Ajuga integrifolia* are traditionally used for treating DM [13].

Antioxidants are known to prevent oxidative stress, thereby treating DM and its complications [14–16]. *Ajuga integrifolia* was confirmed to have well-established in vitro antioxidant activities and had inhibitory concentration, IC50 value of about 18.9 [17], giving hint the plant may have antidiabetic activity. It was screened phytochemically and reported that terpenoides, glycosides, tannins, flavonoids, alkaloids, steroids, phenols, and saponins were most commonly isolated biologically active principles responsible for its medicinal properties [13]. In addition, the roots of *Ajuga integrifolia* have larger amounts of chromium which may be correlated to its use as treatment for DM [18]. Thus, the present study was performed to determine the antidiabetic activity of the root extract and solvent fraction of *Ajuga integrifolia* in mice.

## 2. Materials and Methods

### 2.1. Drugs, Chemicals, and Instruments

Drugs and chemicals used in the study were streptozotocin (Sigma Aldrich, Germany), methanol absolute (Nice Chemical, India), glibenclamide (Julphar pharmaceutical, Ethiopia), trisodium citrate dehydrate (Blulux Laboratories, India), citric acid monohydrate (Lab Tech chemical, India), 40% glucose solution (Reyoung Pharmaceutical, China), sterilized water for injections (Nirman Ltd., India), and distilled water, whereas the instruments used in this study were analytical balance, pH meter, glucometer and test strips (Alliance international, Taiwan), beakers, Whatman filter paper No.1, funnels, glass rod, measuring cylinder, vacuum pump, spatula, pipettes, gavage (oral feeding syringe), animal cages, insulin syringe with needle, oven, and desiccators. Analytical grade drugs, chemicals, and instruments were used.

### 2.2. Collection of Plant Material

Fresh root of *Ajuga integrifolia* was collected from Gondar town in North Gondar, North Ethiopia. The botanical identification and authentication of the plant material was performed by a botanist, and voucher specimens (MA001/2019) were deposited in Herbarium of Biology Department, Faculty of Natural and Computational Science, University of Gondar.

### 2.3. Preparation of Plant Crude Extract and Fractionation

The dirt and particulate matter was removed from the root of *Ajuga integrifolia* by washing rigorously with distilled water. Then, root of *Ajuga integrifolia* was dried under shade at room temperature (20–30°C) with most favorable ventilation. The dried roots of the plant were pulverized into coarse powder by an appropriate electrical miller. The coarse powdered roots of the plant were macerated in 80% methanol in a ratio of 750 g of sample material: 7.5 liters of solvent (w/v) separately. Then, the extract was filtered by using gauze followed by Whatman filter paper No. 1. The marc was remacerated two times with fresh solvent (80% methanol), each for 3 days, and the filtrates so obtained from the sequential macerations were allowed to concentrate removing the methanol solvent by a rotavapor followed by drying in an oven with a temperature of not greater than 40°C, and the aqueous part was removed by lyophilization under reduced pressure. Then, the remaining solvent free extract was kept alone in a desiccator till it was used for the study and the fractionation process in case the extract could contain hygroscopic element.

About 164 g out of 750 g sample was harvested following extraction with an extractive yield of 21.9%. For fractionation, 100 g of methanol crude extract was suspended in a separatory funnel with 400 ml of distilled water. Then, the suspension was shaken by adding 400 ml volume of *n*-hexane. Then, the *n*-hexane layer so formed was poured into a beaker and labeled as “*n*-hexane fraction.” The aqueous remainder was again mixed with same quantity of chloroform and shaken similarly, and the chloroform layer obtained was decanted to a second beaker and labeled as “chloroform fraction” likewise. The remaining aqueous residue was lyophilized to obtain pure aqueous fraction, placed in a third beaker and labeled as “aqueous fraction,” and the *n*-hexane and chloroform fractions were allowed to concentrate in an oven under a temperature set at 40°C. Finally, 12 g hexane and 17 g of chloroform fractions and 2 g of oleaginous jelly matter were obtained with the remaining 69 g being aqueous fraction. All fractions were put in an amber bottle and stored in a fridge till they were going to be used for the experiment.

### 2.4. Experimental Animals

Swiss albino male mice which were healthy weighed 20–30 g and aged 8–12 weeks were used in the study, and healthy female mice having similar weight and age to males were used for the acute oral toxicity study. The mice used for the study were acquired from the Ethiopian Public Health Institute. The mice were stayed in light and dark cycle (12 hours of each cycle) which is a standard condition for laboratory animals and permitted open access to standard pellet diet and water ad libitum. Acclimatization of the mice to the laboratory conditions was performed for 1 week prior to the start of the experiment.

### 2.5. Acute Oral Toxicity Study

According to the limit test of OECD No. 425 guideline, the acute oral toxicity test was performed [19]. One female Swiss albino mouse was fasted for 4 hours in the first day of the test. Then, 2 g/kg of the extract was administered via mouth and was observed rigorously for physical or behavioral changes for one day with special consideration during the first 4 hours. According to the results of the first mouse, another four female mice were recruited and fasted for 4 hours and then given a single dose of 2 g/kg and was observed rigorously in the same manner. A total of 2 weeks was used to observe for development of any signs of toxicity [20]. According to the result of the acute oral toxicity study, the lower, medium, and high doses of the plant extracts were determined.

### 2.6. Grouping and Dosing of Mice

Male mice were used in all mice models (normoglycemic, oral glucose loaded, one dose treated diabetic, and repeated dose treated diabetic mice) because female mice are less sensitive to insulin [21] and STZ compared to male mice [20, 22]. Mice were randomly divided into five groups (6 mice per group) in the normoglycemic, oral glucose loaded, and one dose treated diabetic mice models. Working to the all cases, group 1 used as negative control was treated with 10 ml/kg distilled water (DW). Experimental groups 2, 3, and 4 received 100 mg/kg, 200 mg/kg, and 400 mg/kg root extract of the plant, respectively, whereas group 5 used as positive control was treated with glibenclamide (GLC) 5 mg/kg, which was a standard drug. In single dose of the three solvent root fraction treated diabetic mice model, mice were randomly divided into 11 groups (each group comprised 6 mice). Group 1 (negative control) was treated with 10 ml/kg DW; the 9 experimental groups received solvent fraction of the root extract of the plant (groups 2, 3, and 4 received three different doses of aqueous fraction of the root extract of the plant; groups 5, 6, and 7 received three different doses of *n*-hexane fraction of the root extract of the plant; and groups 8, 9, and 10 received three different doses of chloroform fraction of the root extract of the plant). Group 11 received 5 mg/kg GLC. GLC was used as a standard drug on basis of previous reports on earlier literatures [23, 24].

In repeated daily doses treated diabetic mice, mice were randomly divided into six groups (5 groups of diabetic mice and 1 another group of normal mice, each group comprised of 6 mice). Group 1 was diabetic control treated with 10 ml/kg DW; doses of 100 mg/kg, 200 mg/kg, and 400 mg/kg root extract of plant were given to experimental groups 2, 3, and 4, respectively; group 5 was diabetic positive control treated with 5 mg/kg glibenclamide, whereas group 6 was normal control treated with 10 ml/kg DW.

### 2.7. Measurement of Blood Glucose Level

Blood samples were taken from the tail vein of each mouse by cutting the tip of the tail aseptically in all mice models. Blood glucose level (BGL) was measured by the i-QARE DSW Taiwan glucometer, and measurement was carried out in triplicates so that the average value could be taken. And the reduction of the blood glucose level in percentage was calculated via the following formula: (G_b_ − Gp)/G_b_ × 100, where G_b_ is the BGL at 0 hr (baseline blood glucose level), and Gp is the blood glucose level after treatment.

### 2.8. Induction of Experimental Diabetes Mellitus

Inductions of diabetes were performed by using streptozotocin (STZ). First, STZ was dissolved in 0.1 M cold citrate buffer (pH = 4.5). Dose of 150 mg/kg of the freshly prepared solution was administered intraperitoneally to 16 hr fasted mice. After ½ hr of STZ administration, food and water were permitted freely to the mice. After 6 h of STZ administration, solution of 5% glucose in a quantity of 1 ml/kg was given to the mice for the next 24 hr to prevent death resulting from hypoglycemic shock. Mice were screened for diabetes after 3 days of STZ injection, and fasting BGL > 200 mg/dl was included in the study as diabetic mice [25, 26]. Then, instantly, STZ-induced diabetic mice were assigned randomly into different groups to carry out the experiment. For the maintenance of dry bedding for polyuric diabetic mice, bedding of the cages was changed every 24 hr after STZ injection.

### 2.9. Evaluation of Hypoglycemic Activity of *Ajuga integrifolia* Root Crude Extract in Normoglycemic Mice

Mice fasted for about 16 hr were divided into 5 different groups (each group comprised of 6 mice) at random. For each mouse, baseline BGL was determined prior to treatment (at 0 hr). Then, each mouse's BGL was determined at 1 hr, 2 hr, 4 hr, and 6 hr posttreatment [23, 24].

### 2.10. Evaluation of the Activity of the Crude Extract of *Ajuga integrifolia* Root Extract on Oral Glucose Tolerance in Normoglycemic Mice

Overnight fasted mice were divided into 5 groups (6 mice in each group) at random. Next, group 1 received 10 ml/kg DW; groups 2, 3, and 4 were treated with hydromethanolic root extract of the plant of 100 mg/kg, 200 mg/kg, and 400 mg/kg, respectively, whereas group 5 received 5 mg/kg GLC. Following 30 minutes of after crude extracts, standard drugs, and DW administration, each mouse was fed with 2 g/kg solution of 40% glucose in a quantity of 1 ml/kg. Each mouse's BGL was measured prior to treatment (at 0 minute) as baseline, and then, at 1/2 hr, 1 hr, and 2 hr after glucose administration [24, 27, 28].

### 2.11. Evaluation of Antihyperglycemic Activity of Single Dose of the Extract in STZ-Induced Diabetic Mice

Overnight fasted diabetic mice were arranged into 5 groups (each group comprised of 6 mice) at random. Next, mice were given DW, root crude extract of the plant, and GLC according to grouping stated above. BGL was determined prior to the starting of treatment (at 0 hr) as baseline, and then, following treatment at 2 hr, 4 hr, 6 hr, and 8 hr.

### 2.12. Evaluation of Antihyperglycemic Activity and the Effect on Bodyweight of Repeated Daily Doses of Extract on STZ-Induced Diabetic Mice

Overnight fasted STZ-induced diabetic mice and normal mice were grouped into 6 groups (5 groups of mice having diabetes and 1 group of normal mice, 6 mice in each group) at random. The diabetic control and normal controls were given 10 ml/kg DW; three different doses of root extract were given to diabetic treatment groups, whereas the diabetic positive control group received 5 mg/kg GLC all dosed once daily for 14 days. BGL and bodyweight of the mice were determined prior to starting treatment on the 1^st^ day (baseline), 7^th^ day, and 14^th^ day [27, 28].

### 2.13. Evaluation of Antihyperglycemic Activity of Single Dose of Aqueous, Chloroform, and Hexane Fractions of Extract in STZ-Induced Diabetic Mice

After overnight fasting, mice were divided into eleven groups (*n* = 6) at random: group 1 serving as the diabetic control group receiving 10 ml/kg DW, group 2 receiving aqueous fraction 100 mg/kg of AIRE, group 3 receiving aqueous fraction 200 mg/kg of AIRE, group 4 receiving aqueous fraction 400 mg/kg of AIRE, group 5 receiving hexane fraction 100 mg/kg of AIRE, group 6 receiving hexane fraction 200 mg/kg of AIRE, group 7 receiving hexane fraction 400 mg/kg of AIRE, group 8 receiving chloroform fraction 100 mg/kg of AIRE, group 9 receiving chloroform fraction 200 mg/kg of AIRE, group 10 receiving chloroform fraction 400 mg/kg of AIRE, and group 11 receiving GLC 5 mg/kg, and BGL of every mouse was determined at 0 hr, 2 hr, 4 hr, 6 hr, and 8 hr of AIRE fractions administration.

### 2.14. Evaluation of Antihyperglycemic Activity and the Effect on Bodyweight of Repeated Daily Doses of Aqueous Fraction of Extract in STZ-Induced Diabetic Mice

Overnight fasted STZ-induced diabetic mice were grouped into 6 groups (6 mice per groups) at random. Then, group 1 used as diabetic control received 10 ml/kg DW; groups 2, 3, and 4 used as diabetic test groups received three doses of aqueous fraction (100 mg/kg, 200 mg/kg, and 400 mg/kg, respectively); group 5 diabetic positive control was treated with 5 mg/kg GLC, and group 6 normal control was treated with 10 ml/kg DW once daily for 14 days. At baseline, BGL and bodyweight of the mice were determined prior to starting treatment on the 1^st^ day of treatment (three days after STZ injection) and after that on 7^th^ day and 14^th^ day [24].

### 2.15. Ethical Consideration

This study was performed in accordance with the recommendation for the care and use of laboratory animals [28]. The protocol of the study was approved by the Research and Ethics Committee of Department of Pharmacology, School of pharmacy, University of Gondar, prior to commencing the study with reference number (school of pharmacy 12-112-2012).

### 2.16. Statistical Analysis

The data were presented as mean ± standard error of the mean (M ± SEM). One-way ANOVA followed by Tukey's post hoc multiple comparison test was used to compare means of all parameters among groups and within groups. *p* values <0.05 were set as statistically significant. SPSS version 21 software was used to do statistical analysis.

## 3. Results

### 3.1. Percentage Yield of Plant Material Extraction

From 750 g of the plant material used for extraction, 164 g of dried grey extract was collected after completing the extraction process with a percentage yield of about 21.9% w/w. The extract was more appreciably soluble in water than in organic solvent producing yields of hexane (12%), chloroform (17%), aqueous (69%), and remaining olegenous residue (2%).

### 3.2. Acute Oral Toxicity Study

In the acute toxicity study of *Ajuga integrifolia* root extract (AIRE) at the limit dose of 2000 mg/kg, mortality of mice and any signs of toxicity (behavioral, neurological, autonomic, or physical changes) did not occur during the first day as well as throughout the course of the study. Thus, the median lethal dose (LD50) of the AIRE can be considered as more than 2000 mg/kg.

### 3.3. Hypoglycemic Activity of the Hydromethanolic Root Extract in Normoglycemic Mice

Between groups analysis showed no significant difference in baseline fasting BGL throughout groups. Although 100 mg/kg, 200 mg/kg, and 400 mg/kg AIRE reduced BGL at 4^th^ hr and 6^th^ hr compared to their baseline values, this reduction was not a significant reduction in the three groups given different doses of AIRE at all time points. GLC 5 mg/kg, however, reduced BGL more significantly at 2^nd^ hr, 4^th^ hr, and 6^th^ hr compared to each groups (negative control, three test groups with three different doses of AIRE) and baseline value. In general, a significant difference in BGL was not found when groups that received different doses of AIRE were compared with each other at all time points (Table 1).

### 3.4. Effect of *Ajuga integrifolia* Root Crude Extract on Oral Glucose Tolerance in Normoglycemic Mice

Significant difference in baseline BGL was not found throughout groups prior to the start of DW, AIRE, and GLC administration (Table 2). Between groups analysis revealed that 200 mg/kg and 400 mg/kg AIRE significantly decreased hyperglycemia at the 2^nd^ hr compared to the BGL at 30 minutes and the negative control group, whereas 5 mg/kg GLC showed a significant reduction of hyperglycemia at 1^st^ hr and 2^nd^ hr of glucose administration compared to the negative control group and 100 mg/kg, 200 mg/kg, and 400 mg/kg AIRE-treated groups. A mouse receiving 400 mg/kg crude root extract showed a statistically significant reduction in BGL at 2^nd^ hr compared to 100 mg/kg and 200 mg/kg treated groups. Within groups analysis revealed that oral glucose loading caused a statistically significant increment in BGL after 30 minutes in all groups compared to the baseline fasting BGL regardless of the treatments given. Besides, in all groups including the negative control, there was a significant reduction in BGL at 60 and 120 minutes when compared to the respective BGL at 30 minutes after glucose administration. At 120 minutes, all AIRE-treated groups showed blood glucose reduction to a level comparable to their respective baseline values.

### 3.5. Antihyperglycemic Activity of Single Dose of *Ajuga integrifolia* Crude Root Extract in STZ-Induced Diabetic Mice

Between and within group analysis were performed to notice difference in BGL throughout various groups and time points, respectively (Table 3). Significant difference in baseline fasting BGL was not observed throughout all groups. Likewise, a significant reduction in fasting BGL was not noticed in all AIRE received groups compared to the GLC-treated groups at all time points. When AIRE-treated groups were compared to the negative control, they did not show a statistically significant reduction in BGL at all time points except at the 8^th^ hour. There was a significant reduction of BGL in 200 mg/kg and 400 mg/kg AIRE received groups at 8^th^ hr compared to the 100 mg/kg AIRE received group when within a group comparison was performed. The reduction of fasting BGL in percentage was recorded as 15.14% in 100 mg/kg, 15.8% in 200 mg/kg, and 20% in 400 mg/kg AIRE at the 8^th^ hr compared to the respective baseline fasting BGL. GLC 5 mg/kg reduced BGL significantly at 2^nd^ hr (*p* < 0.01), 4^th^ hr (*p* < 0.001), 6^th^ hr (*p* < 0.001), and 8^th^ hr (*p* < 0.001) compared to the respective baselines.

### 3.6. Antihyperglycemic Activity of Repeated Daily Doses of Crude Extract of *Ajuga integrifolia* Root in STZ-Induced Diabetic Mice

Within groups' analysis of data revealed that fasting BGL was reduced in groups that received crude extract compared to the respective baseline values at all time points. The doses of 100 mg/kg, 200 mg/kg, and 400 mg/kg AIRE reduced fasting BGL with a magnitude of 16.10%, 21.6%, and 26.4% after 7^th^ day of treatment and 18.5%, 24.6%, and 28.8% after 14^th^ day of treatment compared to the respective baseline BGL. The fasting BGL of the diabetic control was increased on the 7^th^ day and 14^th^ day of treatment compared to the baseline value. The fasting BGL of the normal control groups, however, did not exhibit remarkable change throughout the study period. Fasting BGL was significantly reduced in the group that received GLC (*p* < 0.01) at 7^th^ day and (*p* < 0.001) at 14^th^ day with percentage reduction of about 60.80% and 65% at the 7^th^ day and 14^th^ day of treatment, respectively, compared to the baseline values. Between groups analysis discovered that groups that received different doses of AIRE revealed a significant reduction in fasting BGL compared to the diabetic control group at the 7^th^ day and 14^th^ day of treatments, and treatment with GLC showed that there was a significant reduction in fasting BGL (*p* < 0.01) at 7^th^ day and (*p* < 0.001) at 14^th^ day compared to the diabetic control group. When GLC-treated groups were compared with the different extract dose treated groups, there was a significant reduction in the GLC-treated group (*p* < 0.05) compared to 200 mg/kg and 400 mg/kg AIRE, *p* < 0.01 compared to 100 mg/kg AIRE at the 7^th^ day and 14^th^ day, and *p* < 0.001 compared to diabetic control (Table 4).

### 3.7. Effect of the Repeated Daily Doses of the *Ajuga integrifolia* Root Crude Extract on Bodyweight of STZ-Induced Diabetic Mice

In comparison to the normal control, STZ caused a significant loss of bodyweight in the diabetic control at 7^th^ day and 14^th^ day of treatment. According to between groups analysis, it was observed that 100 mg/kg AIRE did not improve bodyweight at the7^th^ day and14^th^ day of treatment compared to the diabetic control, but at 200 mg/kg and 400 mg/kg, AIRE improved significantly the bodyweight of the mice (*p* < 0.05) at the 7^th^ day and (*p* < 0.01) at the 14^th^ day of treatment compared to the diabetic control. The AIRE at these doses improved bodyweight significantly (*p* < 0.01) at 7^th^ day and *p* < 0.001) at 14^th^ day compared to the respective baseline. In comparison to the diabetic control, GLC significantly improved the bodyweight loss of STZ-induced diabetic mice at 7^th^ day and 14^th^ day of treatment (Table 5).

### 3.8. Antihyperglycemic Activity of Single Doses of Three Fractions in STZ-Induced Diabetic Mice

When within group analysis was performed, the difference in reduction of fasting BGL was not significant at all time points compared to both the diabetic control and the respective baseline values of all fractions. The reduction of fasting BGL in the GLC-treated group was significant (*p* < 0.05), with a reduction of 24.19%, 37.8%, 48.4%, and 65.2% at the 2^nd^ hr, 4^th^ hr, 6^th^ hr, and 8^th^ hr compared to their baseline values. It reduced fasting BGL considerably at these time points compared to the diabetic control as well. Between groups analysis revealed that aqueous fraction of *Ajuga integrifolia* at 200 mg/kg and 400 mg/kg showed virtually significant (*p* < 0.05) reduction in BGL by decreasing with 16.8% and 21.10% at the 8^th^ hr compared to hexane and chloroform fractions of the same dose. Compared to both the baseline and negative control values, the doses of 100 mg/kg, 200 mg/kg, and 400 mg/kg chloroform and *n*-hexane fractions revealed no significant difference in BGL (Table 6).

### 3.9. Antihyperglycemic Activity of Repeated Daily Doses of Aqueous Fraction of AIRE in STZ-Induced Diabetic Mice

According to between groups' analysis, groups treated with repeated daily doses of aqueous fractions of 100, 200, and 400 mg/kg AIRE resulted in a considerable BGL reduction at the 7^th^ day and 14^th^ day compared to their baseline values (Table 7). Moreover, there was a statistically significant (*p* < 0.01) and (*p* < 0.001) reduction of BGL in the GLC-treated group at 7^th^ day and 14^th^ day, respectively, compared to baseline values with around 61.30% and 64.10% reduction, respectively.

Significant reduction in BGL was observed in the GLC received group at 7^th^ day and 14^th^ day compared to the diabetic control, and also, BGL was reduced dramatically compared to 100 mg/kg, 200 mg/kg, and 400 mg/kg aqueous fraction at the 7^th^ day and 14^th^ day. The AIRE 200 mg/kg and 400 mg/kg reduced fasting BGL significantly at the 7^th^ day and 14^th^ day compared to the baseline value. Fasting BGL was reduced significantly at doses of 200 mg/kg and 400 mg/kg aqueous fraction of AIR at 7^th^ day and 14^th^ day compared to the 100 mg/kg treated group and the diabetic control (Table 7).

### 3.10. Effect of Repeated Daily Doses of Aqueous Fraction of AIRE on Bodyweight of STZ-Induced Diabetic Mice

Significant increase in bodyweight was not noticed in mice treated with each of the three doses of aqueous fraction of AIRE at 7^th^ day and 14^th^ day of treatment compared to their baseline bodyweight in within group comparison (Table 8). Comparison within the group showed that the GLC-treated group increased bodyweight only slightly on the 14^th^ day of treatment. Bodyweight of mice received 200 mg/kg and 400 mg/kg aqueous fraction but not 100 mg/kg was considerably improved at 7^th^ day and 14^th^ day, with percentage increment of 4.95% and 6.32%, respectively, compared to the baseline. There was no significant change in bodyweight of the normal control compared to its baseline weights.

Bodyweight loss of the diabetic control group was significant at 7^th^ day and 14th day, with percentage decrement of 12% and 19.6% compared to the corresponding baseline bodyweight, respectively. Between groups analysis depicted that bodyweight loss of the diabetic control mice was significant at 7^th^ day and 14^th^ day compared to the aqueous fraction treated groups, normal control group, and GLC-treated group (Table 8).

## 4. Discussion

Diabetes mellitus, being a major global health threat nowadays [29], is seeking for studies to discover new therapeutic agents from various origins. Medicinal plants are among the most common sources and mainstay options of medicines for about 75–80% of world population [30]. In the use of synthetic medicines for the treatment of various diseases, free radicals are often generated in the body which may result in an additional disease. Free radical mediated reactions are terminated by plants and drugs of plant origin that have abundant antioxidants, thereby preventing the body from oxidative damage and stress [30–32]. The antidiabetic activity of hydromethanolic root extract and solvent fractions of *Ajuga integrifolia* root was evaluated in a variety of animal models. In the acute oral toxicity study at the limit dose of 2000 mg/kg *Ajuga integrifolia* root extract, mortality of mice was not seen during the study period. This safety profile is consistent with a report by Tafese et al. and with the toxicity study of leaf extract of the same plant [24]. Therefore, the oral median lethal dose (LD50) of the AIRE can be considered to be more than 2000 mg/kg. No toxicity incidences have been reported related to *Ajuga integrifolia* in particular and the genus *Ajuga* in general so far [24, 33–35].

Induction of diabetes mellitus is most commonly performed by using STZ due to its higher inductive rate and selectivity. It causes pancreatic *β*-cell destruction via DNA alkylation and strand breakage, thereby causing diabetes mellitus [36, 37]. It is similar in structure to glucose and hence competes with glucose for transport via pancreas beta cell membrane transporter GLUT-2 and is given for the laboratory animals in a fasting state to overcome competition by glucose for entry [21, 38]. Diabetes mellitus was effectively induced by administering STZ solution (150 mg/kg) to all groups of mice. This was validated after 72 hours of STZ injection with sustained hyperglycemia.

GLC showed the antihyperglycemic effect in diabetic mice, and literature acknowledged that GLC exerts its effect by selectively blocking the ATP sensitive K^+^ channels (K_ATP_) in the *β*-cells of the pancreas. Then, blockage of KATP results in the influx of Ca^2+^ to cells which, in turn, cause depolarization in the cytosol with subsequent insulin secretion [39]. In this study, there was blood glucose lowering brought about by GLC-induced secretion of insulin from pancreatic *β*-cells; this indicates that a single dose of STZ at 150 mg/kg could not cause absolute destruction of *β*-cells.

A single dose of AIRE ranging from 100 mg/kg to 400 mg/kg and GLC 5 mg/kg were given to mice in the normoglycemic model. In this model, BGL of mice was reduced by different doses of hydromethanolic crude extract of *Ajuga integrifolia* root at the 4^th^ hr and 6^th^ hr compared to the baseline value, but a significant difference was not noticed. When different doses of the hydromethanolic root crude extract, baseline value, and negative control group were compared to GLC, GLC reduced the fasting BGL significantly at 2^nd^ hr, 4^th^ hr, and 6^th^ hr. These findings are almost similar to the antidiabetic activity study reports by Belayneh and Birru [23].

The oral glucose tolerance test is a suitable model to measure and evaluate the insulin action and tissues sensitivity to insulin whose pancreatic release is stimulated by ingestion of glucose. It is usually used to detect persons at risk of prediabetes and diabetes [39, 40]. Compared to the negative control, 100 mg/kg AIRE did not result in a significant (*p* > 0.05) reduction of hyperglycemia at all time point except at the 2^nd^ hr (120 minutes), whereas hyperglycemia was reduced significantly by GLC at the1^st^ (*p* < 0.01) and 2^nd^ (*p* < 0.001) hours. However, AIRE 100 mg/kg, 200 mg/kg, and 400 mg/kg reduced fasting BGL approximately close to baseline and virtually similar to the GLC treated group at 120 minutes. This way modulation of blood glucose is consistent with the reports by Belayneh and Birru [23] and Anitha et al. [41]. It was evident that the BGL of mice reached its climax after 30 minutes of administration, and then, it decreased mildly at 60 minutes and reduced nearly to the normal level after 2 hours of glucose administration. The elevation of blood glucose in this pattern and its reduction at these times is consistent with the one reported by Belayneh and Birru [23]. This blood glucose reduction after 2 hours may be due to the glucose lowering activity of AIRE augmented by metabolic utilization of the already consumed glucose. The increased fasting BGL at 30 minutes can be explained that the glucose oral load was absorbed and reached systemic circulation by this time.

Compared to repeated daily doses at 7^th^ day and 14^th^ day, single dose 200 mg/kg and 400 mg/kg AIRE showed lesser reduction of fasting BGL at 6^th^ hr and 8^th^ hr supporting that the repeated daily doses of AIRE have better antidiabetic activity than the single dose. Significant reduction of fasting BGL was noticed following the repeated daily doses of AIRE after the 7^th^ day and 14^th^ day of treatment compared to both diabetic control and baseline. This finding is almost similar to the antidiabetic activity study reports by Belayneh and Birru [23, 27]. Variation in fasting BGL was significant in normoglycemic mice at the 7^th^ day and 14^th^ day of the study procedure, but a significant increase in fasting BGL was seen in the diabetic control group compared to normal control and repeated daily extract doses treated groups. Compared to baseline, fasting BGL was reduced by GLC, with percentage reduction of 60.8% and 65.2% after 7^th^ day and 14^th^ day of treatment, respectively.

Among the solvent fractions (aqueous, chloroform, and hexane), the single dose of the aqueous fraction revealed by far the most credible antihyperglycemic activity in STZ-induced diabetic mice compared to the baseline, diabetic control groups, chloroform-treated groups, and *n*-hexane-treated groups. The chloroform and *n*-hexane fractions did not demonstrate a noticeable antihyperglycemic activity. This could suggest that common antidiabetic phytochemical constituents such as polyphenols, flavonoids, tannins, and chromium were associated to aqueous polar solvent, water, and likely to be contained in neither chloroform nor hexane [13, 18].

Among the three single different doses of aqueous fractions, AQA 400 mg/kg showed highest blood glucose lowering activity. The investigation implied that the single dose and repeated daily doses of aqueous fraction and repeated daily doses of crude extract have a comparable blood glucose lowering ability. Moreover, in repeated daily dose experiment, the GLC-treated group showed a significant reduction in BGL at 7^th^ day and 14^th^ day compared to groups that received various doses of aqueous fraction and crude extract of *Ajuga integrifolia* root. Weight loss of the mice is often associated to STZ and hyperglycaemic complication [22]. Compared to groups treated by both repeated daily doses of crude extract and aqueous fraction of AIRE, the bodyweight of the diabetic control group was reduced significantly after two weeks of treatment. Compared to the baseline bodyweight of the mice, there was a significant increase in bodyweight of the mice following repeated daily doses of aqueous fraction and crude extract at 14^th^ day. The loss of bodyweight in STZ-induced diabetic mice was significantly improved by GLC (*p* < 0.05) at the 14^th^ day of treatment compared to the diabetic control.

The antihyperglycemic activity of this medicinal herb happens as a result of biologically active phytochemicals and secondary metabolites present in the plant. These phytochemicals include phenolic compounds, alkaloids, terpenoids, flavonoids, tannins, and sterols among others. Therefore, the presence of biologically active phytochemicals may impart the blood glucose lowering effect to *Ajuga integrifolia* because these biologically active phytochemicals are known to lower blood glucose [13, 18, 42].

Antihyperglycemic mechanism(s) of action of *Ajuga integrifolia* might be attributed to potentiating of the effect of insulin either by escalating the release of insulin from *β*-cells of pancreas, increasing the utilization of glucose by peripheral tissue, reducing hepatic gluconeogenesis, inhibiting metabolic degradations of carbohydrates, or by preventing oxidative stress [43]. Nevertheless, the present study recommends more in-depth molecular studies to determine the precise mechanism of *Ajuga integrifolia* to lower blood glucose.

## 5. Conclusion

In the present study, adequate data were generated that uphold the claimed antihyperglycemic activity of the medicinal plant *Ajuga integrifolia* in the community. Hence, it can be concluded that *Ajuga integrifolia*, especially at 200 mg/kg and 400 mg/kg of bodyweight of both the crude extract and aqueous solvent fraction, is remarkably effective against STZ-induced diabetic mice models as well as in the oral glucose loaded mice models, thereby validating its ethnomedicinal usage. As far as the major finding of this investigation is concerned, *Ajuga integrifolia* root extract has antihyperglycemic activities. According to the findings, the use of *Ajuga integrifolia* for treatment of diabetes mellitus by a traditional healer is supported by this study.

## Figures and Tables

**Table 1:** Hypoglycemic activity of AIR crude extract in normoglycemic mice.

Blood glucose level (mg/dl)					
Group	0 hr	1 hr	2 hr	4 hr	6 hr
DW10 mg/kg	70.60 ± 0.707	69.40 ± 1.208	71.20 ± 2.131	72.40 ± 1.860	68.60 ± 1.304
AIRE100 mg/kg	74.00 ± 2.449	75.40 ± 2.135	74.80 ± 1.503	69.20 ± 2.634	67.80 ± 2.088
AIRE200 mg/kg	69.60 ± 1.208	69.60 ± 1.503	70.60 ± 0.872	67.20 ± 0.860	68.40 ± 1.581
AIRE400 mg/kg	72.60 ± 1.568	72.90 ± 1.001	73.80 ± 2.267	69.10 ± 1.778	66.20 ± 1.631
GLC 5 mg/kg	72.40 ± 1.560	55.80 ± 2.985	51.60 ± 2.272^a1b1c1d1*β*1^	43.60 ± 1.568^AzBxCxDx*β*y^	36.60 ± 1.965^AzByCyDy*β*z^

*N* = 6 for each treatment; each value is presented in mean ± SEM. ^A^Compared to the negative control; ^*β*^compared to the baseline blood glucose level; ^B^compared to 100 mg/kg AIRE; ^C^compared to 200 mg/kg AIRE; ^D^compared to 400 mg/kg AIRE.  ^X^*p* < 0.05;^y^*p* < 0.01;  ^z^*p* < 0.001. AIRE, *Ajuga integrifolia* root extract; DW, distilled water; GLC, glibenclamide.

**Table 2:** Oral glucose tolerance test of crude root extract of *Ajuga integrifolia* in the oral glucose loaded mice model.

Blood glucose level (mg/dl)				
Group	0 min	30 min	60 min	120 min
DW10 ml/kg	82.8 ± 2.634	214.6 ± 5.802^*β*z^	157.8 ± 4.164^*β*z3x^	113.8 ± 4.974^3z^
AIRE100 mg/kg	87.6 ± 2.249	211.2 ± 4.994^*β*z^	148.8 ± 4.944^*β*z3y^	89.8 ± 2.437^Ax3z^
AIRE200 mg/kg	82.6 ± 2.731	205.6 ± 5.820^Ax*β*z^	148.6 ± 5.573^Ax*β*z3y^	88 ± 2.530 5^AxBx3z^
AIRE400 mg/kg	73.8 ± 1.625	212.6 ± 7.852^Ax*β*z^	142.8 ± 3.105^Ax*β*z3z^	79.6 ± 4.98^AyBxCx3z^
GLC5 mg/kg	76 ± 2.408	154.4 ± 7.461^AyByCyDy*β*z^	73.6 ± 5.112^AzByCyDy*β*z3y^	61.8 ± 6.715^AxBxCxDx*β*z3z^

*N* = 6 for each treatment; each value is presented in mean ± SEM. ^A^Compared to the negative control; ^*β*^compared to the baseline blood glucose level; ^B^compared to 100 mg/kg AIRE; ^C^compared to 200 mg/kg AIRE; ^D^compared to 400 mg/kg AIRE; ^3^compared to the blood glucose level at 30 minute.  ^x^*p* < 0.05;^y^*p* < 0.01;  ^z^*p* < 0.001. DW, distilled water; AIRE, *Ajuga integrifolia* root extract; GLC, glibenclamide. Time in minute indicates after oral glucose load.

**Table 3:** Effect of single dose AIRE on hyperglycemia in STZ-induced diabetic mice.

Blood glucose level (mg/dl)					
Group	0 hr	2 hr	4 hr	6 hr	8 hr
DW10 ml/kg	391.6 ± 2.249	386.2 ± 5.877	395 ± 1.581	393.8 ± 1.497	395.4 ± 1.600
AIRE100 mg/kg	416 ± 27.817	382 ± 19.514	365 ± 10.124	369 ± 9.894	253 ± 9.160^Ax*β*y^
AIRE200 mg/kg	377.4 ± 21.970	358.4 ± 18.10	343.6 ± 16.336	339.2 ± 12.816	317.6 ± 10.671^AxBx*β*y^
AIRE400 mg/kg	393 ± 11.176	368.6 ± 14.038	362 ± 17.283	349.2 ± 19.523	313.4 ± 8.897^AxBx*β*y^
GLC mg/kg	376.4 ± 8.698	282.6 ± 2.960^Ax*β*y^	197.2 ± 4.224^Ax*β*z^	178.2 ± 2.653^AyBxCxDx*β*z^	168 ± 3.975^az bxcxdx*β*z^

*N* = 6 for each treatment; each value is presented in mean ± SEM. ^A^Compared to the negative control; ^*β*^compared to the baseline blood glucose level; ^B^compared to 100 mg/kg AIRE; ^C^compared to 200 mg/kg AIRE; ^D^compared to 400 mg/kg AIRE.  ^x^*p* < 0.05;^y^*p* < 0.01;  ^z^*p* < 0.001. DW, distilled water; GLC, glibenclamide; AIRE, *Ajuga integrifolia* root extract.

**Table 4:** The activity of repeated daily doses of crude extract of AIRE on hyperglycemia in STZ-induced diabetic mice.

Blood glucose level (mg/dl)					
Group	FBGL at baseline	FBGL after 7 days	FBGL after 14 days	*R* (%) at 7 days	*R* (%) at 14 days
DW10 ml/kg	391.6 ± 2.249	393.8 ± 1.967	397.6 ± 4.226	−0.56	−1.50
AIRE100 mg/kg	416 ± 27.817	349.4 ± 17.657^Ax*β*znx^	338.9 ± 19.530^Ax*β*znx^	16.10	18.50
AIRE200 mg/kg	377.4 ± 21.970	296.6 ± 11.369^Ax*β*znx^	284.4 ± 4.760^Ay*β*znx^	21.40	24.60
AIRE400 mg/kg	393 ± 11.176	288.5 ± 9.963^Ax*β*zny^	279.8 ± 10.856^Ax*β*zny^	26.50	28.80
GLC5 mg/kg	376.4 ± 8.698	147.2 ± 3.056^AyByCxDx*β*yny^	129.4 ± 3.429^AzByCxDx*β*zny^	60.80	65.60
Normal control	77.6 ± 7.987	77.4 ± 2.966	77.2 ± 1.815	0.25	0.51

*N* = 6 for each treatment; each value is presented in mean ± SEM. ^A^Compared to diabetic control; ^*β*^compared to the baseline blood glucose level; ^B^compared to 100 mg/kg AIRE; ^C^compared to 200 mg/kg AIRE; ^D^compared to 400 mg/kg AIRE; ^n^compared to normal control.  ^x^*p* < 0.05;^y^*p* < 0.01;  ^z^*p* < 0.001. DW, distilled water; GLC, glibenclamide; AIRE, *Ajuga integrifolia* root extract; R, reduction.

**Table 5:** The effect of repeated daily doses of *Ajuga integrifolia* root extract on bodyweight in STZ-induced diabetic mice.

Bodyweight (gram)				
Group	Before induction	Baseline	7 days after t/t	14 days after t/t
DW 10 ml/kg	29.80 ± 1.251	27.31 ± 1.140	25.75 ± 1.900	23.09 ± 1.371
AIRE 100 mg/kg	28.25 ± 1.89	26.84 ± 2.331	25.92 ± 2.170	24.74 ± 1.382
AIRE 200 mg/kg	27.92 ± 2.71	25.62 ± 2.120	26.53 ± 1.070^Ax*β*ynx^	26.33 ± 1.080^Ay*β*znx^
AIRE400 mg/kg	28.10 ± 2.092	26.90 ± 1.220	27.25 ± 1.320^Ax*β*ynx^	27.6 ± 1.684^Ay*β*znx^
GLC5 mg/kg	26.80 ± 1.631	25.71 ± 1.933	27.48 ± 2.124^Ay*β*znx^	27.90 ± 2.153^Az*β*znx^
Normal control	30.40 ± 2.360	29.53 ± 2.730	30.10 ± 1.552	29.53 ± 1.447

*N* = 6 for each treatment; each value is presented in mean ± SEM. ^A^Compared to the diabetic control; ^*β*^compared to the baseline blood glucose level; ^B^compared to 100 mg/kg AIRE; ^C^compared to 200 mg/kg AIRE; ^D^compared to 400 mg/kg AIRE; ^*n*^compared to normal control.  ^x^*p* < 0.05;^y^*p* < 0.01;  ^z^*p* < 0.001. DW, distilled water; GLC, glibenclamide; AIRE, *Ajuga integrifolia* root extract; t/t, treatment.

**Table 6:** The activity of single doses of three fractions of AIRE on hyperglycemia in STZ-induced diabetic mice.

Blood glucose level (mg/dl)					
Group	0 hr	2 hr	4 hr	6 hr	8 hr
DW10 ml/kg	391.6 ± 5.03	393.2 ± 3.023	391.6 ± 4.261	388.6 ± 3.487	391.8 ± 2.223
AQA100 mg/kg	415.8 ± 62.311	396 ± 34.095	401.6 ± 10.870	394 ± 5.621	388.4 ± 27.817
AQA200 mg/kg	375.4 ± 52.600	336.2 ± 22.030	316.8 ± 21.224	323.4 ± 19.065	312.7 ± 23.376^AxCxHx*β*x^
AQA400 mg/kg	393 ± 24.900	354.2 ± 18.940	359.4 ± 10.539	334.6 ± 7.004	309.8 ± 5.643^AxCxHx*β*x^
HX100 mg/kg	376.4 ± 8.698	384.8 ± 5.526	372.4 ± 14.590	370 ± 9.560	377.8 ± 11.508
HX200 mg/kg	387 ± 12.892	373.2 ± 16.451	399.4 ± 11.830	413 ± 16.257	378.6 ± 10.722
HX400 mg/kg	395.6 ± 15.045	421.2 ± 16.488	418.6 ± 11.927	394.2 ± 5.544	430.4 ± 24.330
CHL100 mg/kg	385.8 ± 8.470	388.2 ± 3.917	399 ± 9.365	390.2 ± 15.160	397.2 ± 9.754
CHL200 mg/kg	394.2 ± 8.381	405.2 ± 17.080	407.8 ± 24.800	418.6 ± 13.851	401.2 ± 11.770
CHL400 mg/kg	386.8 ± 7.179	380.2 ± 5.324	306 ± 12.779	406.4 ± 8.334	387.8 ± 4.375
GBC5 mg/kg	376.4 ± 19.450	292.6 ± 8.004^Ax*β*x^	240.9 ± 8.213^Ax*β*x^	199.2 ± 8.399^Ay*β*y^	134.6 ± 3.842^Az*β*z^

*N* = 6 for each treatment group; each value is presented in mean ± SEM. ^A^Compared to negative control; ^*β*^compared to baseline; ^B^compared to 100 mg/kg; ^C^compared to chloroform; ^H^compared to hexane.  ^x^*p* < 0.05;^y^*p* < 0.01;  ^z^*p* < 0.001. AQA, aqueous; HX, hexane; CH, chloroform; GLC, glibenclamide.

**Table 7:** The activity of repeated daily doses of aqueous fraction of AIRE against hyperglycemia in STZ-induced diabetic mice.

Blood glucose level (mg/dl)					
Group	Baseline BGL	BGL at 7^th^ day	BGL at 14^th^ day	7^th^ day *R* (%)	14^th^ day *R* (%)
DW10 ml/kg	391.6 ± 5.030	429.2 ± 33.041	489.2 ± 43.101	−9.58	−24.85
AQA100 mg/kg	415.8 ± 62.311	379.19 ± 25.203^Ax*β*xnx^	372.8 ± 26.329^Ax*β*xnx^	7.93	9.43
AQA200 mg/kg	375.4 ± 52.681	311.20 ± 35.181^AyBx*β*xnx^	296.2 ± 36.093^AyBx*β*xnx^	16.76	20.52
AQA400 mg/kg	393 ± 24.990	306.21 ± 20.584^AyBx*β*xnx^	286 ± 21.91^AyBx*β*xnx^	22.13	23.89
GLC5 mg/kg	376.4 ± 19.45	146.6 ± 16.607^AyBzCxDy *β*ynx^	135.2 ± 6.907 ^AzBzCyDy*β*zn1^	61.3.0	64.10
Normal control	82.6 ± 4.231	81.9 ± 2.890	82.8 ± 1.931	0.840	−0.20

*N* = 6 for each group; each value is presented in mean ± SEM. ^A^Compared to diabetic control; ^*β*^compared to baseline; ^B^compared to 100 mg/kg; ^C^compared to 200 mg/kg; ^D^compared to 400 mg/kg; ^n^compared to normal control.  ^x^*p* < 0.05;^y^*p* < 0.01;  ^z^*p* < 0.001. GLC, glibenclamide; AQA, aqueous; DW, distilled water; *R*, reduction.

**Table 8:** Effect of repeated daily doses of aqueous fraction of AIR on bodyweight in STZ-induced diabetic mice.

Bodyweight (gram)				
Group	Before induction	Baseline	After 7 days	After 14 days
DW10 ml/kg	29.34 ± 1.516	26.4 ± 0.894	23 ± 2.915^Gx*β*xnxBxCxDx^	21.2 ± 1.781^Gx*β*xnxBxCxDx^
AIRE100 mg/kg	30.8 ± 3.271	26.6 ± 2.074	25.3 ± 1.732	26.2 ± 1.924
AIRE200 mg/kg	27 ± 2.739	24.2 ± 1.924	24.8 ± 2.550^*β*x^	25.4 ± 2.387^*β*x^
AIRE400 mg/kg	27.4 ± 2.074	25.3 ± 1.673	25.6 ± 1.817^*β*x^	26.9. ± 1.789^*β*x^
GLC5 mg/kg	28.4 ± 4.615	26.8 ± 1.789	27.84 ± 1.483	28.7 ± 1.516^*β*x^
Normal control	28.2 ± 3.033	28.9 ± 2.302	29 ± 2.121	29.4 ± 2.074

*N* = 6 for each group; each value is presented in mean ± SEM. ^G^Compared to 5 mg/kg GLC; ^*β*^compared to the baseline value; ^B^compared to 100 mg/kg AIRE; ^C^compared to 200 mg/kg; ^D^compared to 400 mg/kg AIRE; ^n^compared to normal control.  ^x^*p*=0.05. AIRE, *Ajuga integrifolia* root extract; GLC, glibenclamide; DW, distilled water.

## Data Availability

The datasets used to support the findings of this study are available from the corresponding author upon request.

## Abbreviations

ANOVA: Analysis of variance
AIRE: *Ajuga integrifolia* root extract
BGL: Blood glucose level
DM: Diabetes mellitus
DW: Distilled water
GLC: Glibenclamide
LD50: Median lethal dose
OECD: Organization for Economic Cooperation and Development
STZ: Streptozotocin
SPSS: Statistical Package for Social Science
USD: United States Dollar.

## References

[1] WHO (2006). *Definition and Diagnosis of Diabetes Mellitus and Intermediate Hyperglycaemia: Report of a WHO/IDF Consultation*.

[2] Tiwari N., Thakur A. K., Kumar V., Dey A., Kumar V. (2014). Therapeutic targets for diabetes mellitus: an update. *Clinical Pharmacology and Biopharmaceutic*.

[3] ADA (2014). Diagnosis and classification of diabetes mellitus. *Diabetic Care*.

[4] Okur M. E., Karantas I. D., Siafaka P. I. (2017). Diabetes Mellitus: a review on pathophysiology, current status of oral pathophysiology, current status of oral medications and future perspectives. *ACTA Pharmaceutica Sciencia*.

[5] International Diabetes Federation (2019). *Diabetes Atlas*.

[6] Piero N. M., Murugi N. J., Mwiti K. C., Mwenda M. P. (2012). Pharmacological management of diabetes mellitus. *Asian Journal of Biochemical and Pharmaceutical Research*.

[7] Marles R. J., Farnsworth N. R. (1995). Antidiabetic plants and their active constituents. *Phytomedicine*.

[8] Meresa A., Gemechu W., Basha H. (2017). Herbal medicines for the management of diabetic mellitus in Ethiopia and Eretria including their phytochemical constituents. *American Journal of Advanced Drug Delivery*.

[9] Seifu A. (2017). *Bioprospecting Potential of Ajuga Integrifolia for Access and Benefit Sharing*.

[10] Assefa F. (2013). Antidiabetic activity of ajuga remota benth (harmegusa) leaves in streptozotocin induced diabetic rats.

[11] Chekole G. (2017). Ethnobotanical study of medicinal plants used against human ailments in Gubalafto District, Northern Ethiopia. *Journal of Ethnobiology and Ethnomedicine*.

[12] Bekele T., Hymete A., Tadesse M., Mekonnen Y. (2008). *Antidiabetic Activity and Phytochemical Screening of Crude Extracts of Stevia rebaudiana Bertoni and Ajuga Remota Benth Grown in Ethiopia on Alloxan-Induced Diabetic Mice*.

[13] Bekeri D., Adane L., Mamo F. (2018). Phytochemical investigation and isolation of compounds from ajuga integrifolia root extract. *World Journal of Chemistry*.

[14] Asmat U., Abad K., Ismail K. (2016). Diabetes mellitus and oxidative stress-a concise review. *Saudi Pharmaceutical Journal*.

[15] Pérez-Matute P., Zulet M. A., Martínez J. A. (2009). Reactive species and diabetes: counteracting oxidative stress to improve health. *Current Opinion in Pharmacology*.

[16] Robertson R. P. (2004). Chronic oxidative stress as a central mechanism for glucose toxicity in pancreatic islet beta cells in diabetes. *Journal of Biological Chemistry*.

[17] Keshebo D. L., Washe A. P., Alemu F. (2016). Determination of antimicrobial and antioxidant activities of extracts from selected medicinal plants. *American Scientific Research Journal for Engineering, Technology, and Sciences (ASRJETS)*.

[18] Pala A., Jadona M., Katarea Y. K. (2011). Ajuga bracteosa wall: a review on its ethnopharmacological and phytochemical studies. *Der Pharmacia Sinica*.

[19] OECD/OCDE (2008). *OECD Guideline for the Testing of Chemichals: Acute Oral Toxicity; Up-And-Down Procedure (UDP)*.

[20] Deeds M. C., Anderson J. M., Armstrong A. S. (2011). Single dose streptozotocin-induced diabetes: considerations for study design in islet transplantation models. *Laboratory Animals*.

[21] Vital P., Larrieta E., Hiriart M. (2006). Sexual dimorphism in insulin sensitivity and susceptibility to develop diabetes in rats. *Journal of Endocrinology*.

[22] Furman B. L. (2015). Streptozotocin-induced diabetic models in mice and rats. *Current Protocols in Pharmacology*.

[23] Belayneh Y. M., Birru E. M. (2018). Antidiabetic activities of hydromethanolic leaf extract of calpurnia aurea (ait.) benth. Subspecies aurea (fabaceae) in mice. *Evidence-Based Complementary and Alternative Medicine*.

[24] Tafesse T. B., Hymete A., Mekonnen Y., Tadesse M. (2017). Antidiabetic activity and phytochemical screening of extracts of the leaves of Ajuga remota Benth on alloxan-induced diabetic mice. *BMC Complementary and Alternative Medicine*.

[25] Tamiru W., Engidawork E., Asres K. (2012). Evaluation of the effects of 80% methanolic leaf extract of Caylusea abyssinica (fresen.) fisch. & Mey. on glucose handling in normal, glucose loaded and diabetic rodents. *BMC Complementary and Alternative Medicine*.

[26] Baquer N. Z., Kumar P., Taha A., Kale R., Cowsik S., McLean P. (2011). Metabolic and molecular action of Trigonella foenum-graecum (fenugreek) and trace metals in experimental diabetic tissues. *Journal of Biosciences*.

[27] Belayneh Y. M., Birhanu Z., Birru E. M., Getenet G. (2019). Evaluation of in vivo antidiabetic, antidyslipidemic, and in vitro antioxidant activities of hydromethanolic root extract of Datura stramonium L.(Solanaceae). *Journal of Experimental Pharmacology*.

[28] Clark J. D., Gebhart G. F., Gonder J. C., Keeling M. E., Kohn D. F. (1997). The 1996 guide for the care and use of laboratory animals. *ILAR Journal*.

[29] Sen S., Chakraborty R., Sridhar C., Reddy Y. S., De B. (2010). Free radicals, antioxidants, diseases and phytomedicines: current status and future prospect. *International Journal of Pharmaceutical Sciences Review and Research*.

[30] Prabhakar P. K., Doble M. (2008). A target based therapeutic approach towards diabetes mellitus using medicinal plants. *Current Diabetes Reviews*.

[31] Sabu M. C., Kuttan R. (2002). Anti-diabetic activity of medicinal plants and its relationship with their antioxidant property. *Journal of Ethnopharmacology*.

[32] Cocquyt K., Cos P., Herdewijn P., Maes L., Van den Steen P. E., Laekeman G. (2011). Ajuga remota Benth.: from ethnopharmacology to phytomedical perspective in the treatment of malaria. *Phytomedicine*.

[33] El Hilaly J., Lyoussi B. (2002). Hypoglycaemic effect of the lyophilised aqueous extract of Ajuga iva in normal and streptozotocin diabetic rats. *Journal of Ethnopharmacology*.

[34] Hailu W., Engidawork E. (2014). Evaluation of the diuretic activity of the aqueous and 80% methanol extracts of Ajuga remota Benth (Lamiaceae) leaves in mice. *BMC Complementary and Alternative Medicine*.

[35] Radenković M., Stojanović M., Prostran M. (2016). Experimental diabetes induced by alloxan and streptozotocin: the current state of the art. *Journal of Pharmacological and Toxicological Methods*.

[36] Etuk E. U. (2010). Animals models for studying diabetes mellitus. *Agriculture and Biology Journal of North America*.

[37] Rees D. A., Alcolado J. C. (2005). Animal models of diabetes mellitus. *Diabetic Medicine*.

[38] Hilakivi-Clarke L. A., Wozniak K. M., Durcan M. J., Linnoila M. (1990). Behavior of streptozotocin-diabetic mice in tests of exploration, locomotion, anxiety, depression and aggression. *Physiology & Behavior*.

[39] Malaisse-Lagae F. R., Malaisse W. J. (1971). Stimulus-secretion coupling of glucose-induced insulin release. III. Uptake of ^45^calcium by isolated islets of Langerhans. *Endocrinology*.

[40] Andrikopoulos S., Blair A. R., Deluca N., Fam B. C., Proietto J. (2008). Evaluating the glucose tolerance test in mice. *American Journal of Physiology-Endocrinology and Metabolism*.

[41] Anitha M., Sakthidevi G., Muthukumarasamy S., Mohan V. R. (2012). Effect of Cynoglossum zeylanicum (Vehl ex Hornem) Thunb. ex Lehm on oral glucose tolerance in rats. *Journal of Applied Pharmaceutical Science*.

[42] Prabhakar P. K., Doble M. U. (2008). Mechanism of action of medicinal plants towards diabetes mellitus-a review. *Recent Progress in Medicinal Plants*.

[43] Jung M., Park M., Lee H. C., Kang Y. H., Kang E. S., Kim S. K. (2006). Antidiabetic agents from medicinal plants. *Current Medicinal Chemistry*.

